# Containment Practices for Arthropods Modified with Engineered Transgenes Capable of Gene Drive Addendum 1 to the Arthropod Containment Guidelines, Version 3.2

**DOI:** 10.1089/vbz.2021.0035

**Published:** 2022-01-13

**Affiliations:** ADDRESS: 241 18th Street South, Suite 501 Arlington, VA 22202, USA

**Keywords:** guidelines, arthropod, containment, gene drive, laboratory safety, vector-borne disease, risk assessment

## Abstract

Responsible conduct of research is a cornerstone of rigorous scientific discovery. Institutional committees, independent advisory panels, and expert steering groups are among the frameworks in academia meant to provide guidance and assurances that research activities do not result in harm to the environment, research staff, or public safety. For research involving arthropods of public health importance, several documents currently exist to guide investigators in methodologies to consider for reducing risks from arthropod escape. However, to date, there has been no standardized set of recommendations on containment practices for arthropods modified with engineered transgenes capable of gene drive. This document is meant to serve as a practical reference to fill that gap. Recommendations outlined here address containment considerations when a risk assessment indicates a possibility of establishment of a new arthropod vector species or genetically modified arthropods in the local environment.

## Intent

The National Institutes of Health (NIH) guidelines for research with recombinant or synthetic nucleic acids provide containment recommendations for work with genetically modified (GM) microorganisms, plants, and large animals, but offer no specific guidance for the containment of arthropods (U.S. National Institutes of Health 1984). A desire to fill this gap provided the impetus for the initial development of the Arthropod Containment Guidelines (ACG) sponsored by the American Committee of Medical Entomology (ACME) in 2003 (American Committee of Medical Entomology; American Society of Tropical Medicine and Hygiene 2003).

**Table 1.** Table of Contents

Abstract...................................................................................................................................................................................	4
Intent ......................................................................................................................................................................................	4
Table 2. Glossary of Terms...................................................................................................................................................	5
Table 3. Acronyms.................................................................................................................................................................	7
Risk Assessment of Gene Drive Strains................................................................................................................................	7
Introduction to performing a risk assessment...................................................................................................................	7
Table 4. Examples of risk assessment questions and considerations regarding accidental escape of arthropods containing gene drives................................................................................................................................................	8
General considerations.......................................................................................................................................................	8
Research conducted where the arthropod species is not found but could become established......................................	9
Research conducted where the arthropod species is found..............................................................................................	9
Reducing and managing risks............................................................................................................................................	10
Risk considerations for laboratories exchanging strains...................................................................................................	10
Table 5. Overview of Arthropod Containment Levels (ACL 1–4) and Biosafety Containment Levels (BSL 1–4) to Include Arthropod Containment Level-2+ presented in this addendum..................................................................	11
Arthropod Containment Levels (ACL) 2+ Recommendations.............................................................................................	10
Introduction to ACL 2+ recommendations........................................................................................................................	10
Facility design and layout..................................................................................................................................................	12
Location of arthropods...................................................................................................................................................	12
Insectary doors................................................................................................................................................................	12
Insectary windows..........................................................................................................................................................	12
Interior surfaces..............................................................................................................................................................	12
Vacuum systems.............................................................................................................................................................	12
Source and harborage reduction.....................................................................................................................................	12
Equipment and supply storage.......................................................................................................................................	12
Plumbing and floor drains..............................................................................................................................................	12
Electrical fixtures............................................................................................................................................................	12
Sink.................................................................................................................................................................................	12
Illumination....................................................................................................................................................................	12
Heating, ventilation, and air conditioning.....................................................................................................................	12
Facility compliance monitoring.....................................................................................................................................	13
Procedures: access/security................................................................................................................................................	13
Security...........................................................................................................................................................................	13
Access restrictions..........................................................................................................................................................	13
Notification and signage.................................................................................................................................................	13
Prevention of accidental dispersal outside the research laboratory by attachment on persons...................................	13
Procedures: handling/manipulation....................................................................................................................................	13
Procedure design............................................................................................................................................................	13
Routine decontamination................................................................................................................................................	13
Special arthropod handling containers and areas..........................................................................................................	13
Isolation of arthropods...................................................................................................................................................	14
Safe transport in the laboratory.....................................................................................................................................	14
Primary container identification and labeling.................................................................................................................	14
Primary container cleaning, disinfestation.....................................................................................................................	14
Disposal of arthropods....................................................................................................................................................	14
Disposal of consumable supplies....................................................................................................................................	14
Prevention of accidental dispersal outside the research laboratory through sewer system.........................................	14
Movement of equipment.................................................................................................................................................	14
Sterilization equipment....................................................................................................................................................	14
Laboratory sharps............................................................................................................................................................	15
Pest exclusion program...................................................................................................................................................	15
Procedures: monitoring/training..........................................................................................................................................	15
Institutional biosafety committee and institutional animal care and use committee approvals...................................	15
Safety manual..................................................................................................................................................................	15
Training............................................................................................................................................................................	15
Monitoring for unintentional arthropod escape..............................................................................................................	15
Escaped arthropod handling............................................................................................................................................	15
Escaped arthropod reporting...........................................................................................................................................	15
Record keeping of escaped arthropods...........................................................................................................................	16
Personal protective equipment........................................................................................................................................	16
Other procedural considerations.........................................................................................................................................	16
Containment director.......................................................................................................................................................	16
Large-cage indoor trials..................................................................................................................................................	16
Emergency planning........................................................................................................................................................	16
Shipping...........................................................................................................................................................................	16
Author Disclosure Statement..................................................................................................................................................	17
Funding Information...............................................................................................................................................................	17
References...............................................................................................................................................................................	17
Appendix A1. Addendum 1 Drafting Committee Members.................................................................................................	17
Appendix A2. Addendum 1 Drafting Process.......................................................................................................................	17

**Table 2.** Glossary of Terms

Term	Definition
Arthropod	An invertebrate animal, for example, mosquitoes and ticks belonging to the phylum Arthropoda
Arthropod containment level (ACL)	Designations (ACL-1 to ACL-4) used to describe arthropod handling practices, safety equipment, and facilities recommended for preventing the escape of arthropods. Level designation is based on assessed risk of harm due to the research procedures and harm from escape.
Autonomous gene drive	Gene drive (natural or engineered) comprising a single genetic unit that contains all the components (*e.g.*, nucleases, transposase, toxin, antitoxin) necessary for its function as a gene drive system (Alphey et al. 2020).
Biosafety level (BSL)	Designations (BSL-1 to BSL-4) used in a laboratory setting to identify the measures needed to protect workers, the environment, and the public from pathogens of veterinary and medical importance. Level designation is based on assessed risk of harm due to the research procedures as described in the Centers for Disease Control Biosafety in Microbiological and Biomedical Laboratories (U.S. Department of Health and Human Services, Public Health Service, Centers for Disease Control and Prevention, National Institutes of Health 2020).
Comparator	An unmodified organism to which a comparison is made.
Conspecific	Of the same species.
Escapee	An arthropod present outside of a secure facility or its intended containment.
Exotic	Organisms not found in a specific environment; nonindigenous species.
Facility	A building, room, or suite of rooms designed and utilized for a stated purpose, for example, for scientific research or arthropod containment.
Fecundity	The ability to produce offspring.
Fitness	The ability of an organism to mate, survive, and produce viable offspring.
Gene drive	A phenomenon of biased inheritance in which the prevalence of a genetic element (natural or synthetic) or specific alternative form of a gene (allele) is increased, even in the presence of some fitness cost. This leads to the preferential increase of a specific genotype that may determine a specific phenotype from one generation to the next, and potentially spread throughout a population (Alphey et al. 2020).
Heterozygous	Having two different alleles of a particular gene or genes
High-frequency threshold	When the number of arthropods carrying an engineered transgene must exceed a substantial fraction of the overall local population to display the process of gene drive.
Homozygous	Having two of the same form, or allele, of a particular gene(s).
Indigenous	Organisms naturally found in a specific environment; not exotic.
Infrastructure	The basic physical and organizational structures and facilities (*e.g.*, buildings, power supplies, water supply, autoclaves, trained staff, and ethical review committees) needed for the operation of the research.
Institutional animal care and use committee	A committee responsible for reviewing institutional research conducted by its faculty, students, or staff that involves the use of vertebrate animals with a goal of maintaining animal welfare standards.
Institutional biosafety committee	A committee responsible for reviewing institutional research conducted by its faculty, students, or staff that involves the use of recombinant or synthetic DNA in combination with biological organisms or agents.
Laboratory	A structure, such as a room, suite of rooms, or building, equipped for scientific experiments.
Laboratory sharps	Instruments or waste with sharp edges that can puncture or cut handlers or regular waste container bags.
Low-frequency threshold	When the number of arthropods carrying an engineered transgene can be very small compared with the overall local population and still display the process of gene drive.
Organism	A living being; an individual animal, plant, or single-celled life form.
Pathogen	A microorganism that can cause disease in humans, animals, and/or plants.
Phenotype	The physical appearance or set of observable characteristics of an organism resulting from the interaction between the individual organism's genotype and the environment.
Population	Organisms of a given species living in a specific geographic location that are capable of interbreeding.
Refractory	Resistant to an infection with a particular pathogen.
Refractory phenotype	Reduced vectorial competence relative to an unmodified strain.
Risk	Potential for loss or harm.
Risk assessment	The process of ascertaining the probability of loss or harm and gauging the severity of potential adverse effects.
Self-limiting gene drive	Approaches where the genetic modification will not pass on indefinitely through subsequent generations, even in the absence of mutations or heritable resistance. For gene drive, this will typically mean that the allele frequency of the gene drive will initially increase (the gene drive phenomenon) but later decrease, eventually disappearing. Also known as self-exhausting drives or approaches (Alphey et al. 2020).
Split gene drive	In a split gene drive, the necessary components for gene drive are split between two (or more) genetic loci. If one (or more) of these components is incapable of gene drive even in the presence of the others, this is a nonautonomous gene drive. If, in the presence of the other elements, each element can exhibit gene drive (“transcomplementing split gene drive”) the components in suitable combination may comprise an autonomous gene drive (q.v. Alphey et al. 2020).
Strain	A subtype of a particular species that has a common geographic or genetic origin. This also applies to pathogens of various genetic compositions.
Suppression phenotype	A reduced capacity to produce reproductively capable offspring.
Transgene	A DNA sequence or gene that is introduced into an organism through genetic engineering.
Transgenic	Of, relating to, or containing DNA transferred into an organism using genetic engineering.
Vector	An organism that carries and transmits an infectious pathogen.
Vectorial capacity	Overall ability of an organism to successfully transmit a pathogen in a natural setting.
Vector competence	Ability of an organism to become infected with a pathogen and transmit that pathogen in an experimental setting.
Wild type	Organisms of the same species that have not been genetically modified.

**Table 3.** Acronyms

ACG	Arthropod containment guidelines
ACL	Arthropod containment level
ACME	American Committee of Medical Entomology
BSL	Biosafety containment level
FNIH	Foundation for the National Institutes of Health
GM	Genetically modified
PPE	Personal protective equipment
SOP	Standard operating procedure

The most recently published version of the ACG (ACG v3.2) continues to be an important and valuable document for biosafety professionals and researchers (American Committee of Medical Entomology; American Society of Tropical Medicine and Hygiene 2019). A Foundation for the National Institutes of Health (FNIH) survey of biosafety professionals published in 2020 found that 67% (*n* = 50) used the ACG for GM and non-GM insect containment decision making; however, although the ACG v3.2 considers transgenic arthropods, the guidelines do not differentiate between gene drive and nongene drive modifications (O'Brochta et al. 2020). In this regard, the same survey revealed that 61% (*n* = 46) of the respondents felt that existing guidance from any current organization/committee was inadequate for making risk assessments and containment decisions regarding arthropods modified with engineered transgenes capable of gene drive. Consequently, research groups studying gene drive-modified arthropods have developed their own blend of containment measures that are at times stricter than current ACG v3.2 Arthropod Containment Level 2 (ACL-2) recommendations, which outline guidelines for arthropods containing transgenes with low potential for gene drive or, in other cases, do not adhere to even the basic containment measures recommended for ACL-1, which applies to nontransgenic arthropods.

To address a recognized need for standard guidance, ACME, with the support of FNIH, convened an advisory group (see Appendix 1) tasked with developing recommendations for containment and best practices in research with arthropods containing engineered transgenes capable of gene drive. Gene drive is a phenomenon of biased inheritance in which the prevalence of a genetic element (natural or synthetic) or specific alternative form of a gene (allele) is increased, even in the presence of some fitness cost. This leads to the preferential increase of a specific genotype that may determine a specific phenotype from one generation to the next, and potentially spread throughout a population (Collins et al. 2016, Alphey et al. 2020). Considering methods currently under development, gene drive can be used to suppress or modify wild populations.

This addendum to the ACG v3.2 is the resulting product of the advisory group based on the subject matter expertise of its membership and information gathered by informal consultation among other subject matter experts (see Appendix 2). The recommendations outlined represent the position of ACME as an organization, not that of all individual members nor their institutions.

This addendum complements current ACG v3.2 ACL-2 recommendations to provide further guidance, termed ACL-2+, for best practices by investigators and associated teams for handling transgenic arthropod vectors with engineered transgenes capable of gene drive.

These ACL-2+ recommendations build upon existing ACG v3.2 ACL-1 and ACL-2 recommendations. The application of these recommendations to any laboratory must be based on a rigorous risk assessment. Although recommendations outlined in this addendum are focused on containment of arthropods containing engineered transgenes capable of gene drive, ACL-2+ recommendations may also be appropriate for the containment of unmodified arthropods where risk assessments indicate a possibility of establishment of escaped vector species currently not present in the local environment. The intention is to provide recommendations for preventing arthropod escape, thereby protecting the health of laboratory personnel conducting the research, other members of the research institution, and the public, as well as protecting animal health and the natural ecosystem surrounding the institution.

The principles of risk assessment, specific practices, and equipment outlined in this addendum are focused on indoor laboratory research involving arthropods of public health importance but could apply to arthropods of agricultural importance. Arthropods considered include insects (Diptera—mosquitoes, tsetse flies, black flies, sand flies, and midges; Hemiptera—kissing bugs; Phthiraptera—lice; Siphonaptera—fleas), and arachnids (Acari—ticks, mites). The diversity of these organisms and their complex life cycles often mean that procedures and practices for safe containment of arthropods target genera, or be life stage-specific and/or species-specific. Conversely, although it is recognized that a wide range of technologies are available to engineer transgenes that result in gene drive, and technology should be part of risk assessment, the recommendations outlined in this addendum are not technology specific but rather focus on protection goals and biosafety outcomes. It should also be recognized that methods employed by investigative teams to meet these recommendations may vary.

Principles of responsible research include respect for the wider public. The ACME and FNIH, through these ACL-2+ recommendations, will foster responsible research practices among the global scientific community and its trainees. This can serve to enhance public safety confidence and methodology for scientists to conduct research. In this spirit, investigators whose work falls under these guidelines should consider using appropriate means as per institutional and/or regulatory processes for public engagement.

## Risk Assessment of Gene Drive Strains

### Introduction to performing a risk assessment

Before conducting research with arthropods containing engineered transgenes capable of gene drive, users of these guidelines should understand the principles of risk assessment. This is a process of evaluating possible harmful outcomes to identified protection goals, such as human and animal health as well as the local environment (often referred to as the “receiving environment”) because of the proposed research activities. The outcome(s) should be considered in comparison with an appropriate “comparator,” for example, an unmodified arthropod of the same or related species. Guidance on conducting a risk assessment for GM organisms is available and recommended for those not familiar with the process (*e.g.*, see figure 1 in Convention on Biodiversity 2016).

The risk assessment process conducted to determine which ACL is most appropriate may vary across research activities, investigative teams, and/or research facilities dependent on the expertise of the research team and/or existing capacity of the institution (*i.e.*, biosafety committees, etc.); however, these guidelines on risk identification have the objective of ensuring a basic approach to hazard identification and probability [likelihood]. Normally this process is conducted by the one proposing the research and approved by the local biosafety or equivalent committee.

Fundamentally, the process of risk assessment for research under biological containment includes reflecting on several questions that are derived from the goals of the proposed research activities in conjunction with the need to assure human (researchers and wider public) and animal health as well as environmental safety in and around the facility. These include: “What potential harm could result to laboratory staff, other individuals, the community, and the environment from an accidental escape of these research organisms? What events could lead to these harms occurring and what procedures would need to fail? How likely is it that these events or failures would occur? Does the research organism in question pose risks that are significantly different from an unmodified comparator under an escape scenario? ‘What would be the severity of any potential harm?’ How could risks be reduced and harms minimized through specific research design and procedures? What processes are in place to engage/address concerns of the local public in response to arthropod escape?” These questions inevitably consider values such as whether possible outcomes are acceptable (Table 4).

**Table 4.** Examples of Risk Assessment Questions and Considerations Regarding Accidental Escape of Arthropods Containing Gene Drives

Select questions	Example considerations
1. Could the experimental arthropod become established in the local environment?	Is there evidence that the same species is already present in the local environment? How may the life cycle of the experimental arthropod and environmental conditions factor into survivability and establishment? Is the climate suitable? Could the arthropod persist through cold/dry/hot periods? Are there related species that could be potential recipients of the altered gene (probability of mating success)? Are there suitable blood meal host(s) of the experimental arthropod currently located in the local environment (probability of survival)?
2. What is the technology that results in gene drive?	Is it an autonomous gene drive? A split drive? Or other molecular safeguards or forms of self-limiting drive?
3. Has the ability of the experimental arthropod to serve as a vector been altered?	Is there reason to believe that the alteration could reduce or increase the probability of transmitting (under laboratory conditions) pathogen(s) that the arthropod is known to vector? Is there reason to believe that the alteration could increase the transmission of other pathogens?
4. Has the fitness of the experimental arthropod been modified?	What is the effect on lifespan? Reproductive capacity? Host-feeding preference? Sex ratio? Blood-feeding frequency? Would these changes negatively or positively affect fitness?
5. Can the arthropod be identified or removed from the local environment in the case of an escape?	What is the susceptibility of the modified arthropod to insecticides or other means of remediation? Are there unique markers or assays to differentiate escapees from wild-type individuals or related cryptic species? Is an emergency response plan in place for potential escapees? Are trapping and control methods required to monitor presence and eliminate the escaped arthropod from the local environment readily available for immediate implementation?
6. What are the projected public health, animal health, and environmental impacts of an escaped organism if remediation is not possible?	Expanded range of natural distribution/territory? Are there anticipated adverse effects on pollinators or other beneficial/valued nontarget organisms?
7. Are there environmental hazards (flood zone, hurricanes, and tornados) at the facility location that should be considered?	What are the parameters, for example, a breakdown in secure areas, that may lead to an escape of the altered arthropods?
8. Are there notification/communication plans related to a potential arthropod escape?	Is there a need to establish a crisis communication plan (timeliness of the notification process, and who should be notified, *e.g.*, at what level of notification required)? Are institutional processes in place for immediate engagement with the local public if this was deemed necessary?

Many of the risks that may be identified during an assessment may not have quantitative estimates available to gauge likelihood of occurrence and/or the degree of harm that might be realized. The risk assessment process requires an informed judgment referencing not only existing information in the public domain but also others' experience with similar research procedure scenarios. This might include taking a weight of evidence approach that considers the combined breadth of experience with research using similar organisms, procedures, and scenarios.

### General considerations

Gene drive methods currently under development aim either to reduce the numbers of target arthropod vectors (suppression phenotype) or reduce their capacity to transmit one or more pathogens (refractory phenotype), though other forms of population alteration phenotypes may be devised. Although the emphasis of these guidelines is on arthropods of medical importance, with the exception of the risk assessment portion, which emphasizes effects on human and animal health, the specific containment recommendations are generally applicable to other arthropods as well. These include those of agricultural, cultural, and ecological importance. Strains that demonstrate reduced fecundity (suppression) or diminished vectorial capacity (refractory) may be expected to present reduced risk to human and animal health compared with the unmodified form in the event of escape and establishment in the wild. However, several possible hazardous outcomes of arthropod escape should be considered in risk assessment. These include, but are not limited to, (1) establishment in the environment of an exotic species or transgene that could reasonably be expected to cause harm to protection goals; (2) an increase in the capacity of the wild population to transmit pathogens, resulting in an increase in animal or human disease; (3) potential migration of another vector species into now unoccupied niches (in the case of suppression), resulting in an increase in human or animal disease; and (4) the evolution of pathogen strains that can evade the refractory phenotypes (in the case of refractory approaches), resulting in an increase in the transmission or severity of human or animal disease.

Gene drive transgenes that contain the components necessary to drive a new phenotype into a population in a single genetic unit are considered autonomous. In contrast, split drives, in which two or more assorting genetic factors are necessary to create drive, have reduced potential for sustained gene drive due to the independent segregation of these components. Other approaches are possible to limit the spread of gene drives either spatially or temporally (localizing and/or self-limiting drives). It is impossible to anticipate all the applications of engineered driving transgenes and the techniques that might be used to modify arthropods to express genes that drive. Therefore, the emphasis of the ACG risk assessment is on the phenotype of strains and potential environments into which they could escape.

Risk assessment should consider whether the gene drive system is one that could spread from a low starting frequency (low threshold drives; the escape of just a few individuals into conspecific populations) or whether a relatively high frequency threshold must be crossed for sustained gene drive to occur. It is important to recognize that even for gene drive approaches that require a high frequency threshold to spread, intermating among unmodified or wild type and gene drive individuals in small subpopulations might establish the gene drive phenotype.

Simple descriptions of the intended gene drive phenotypes, however, do not fully capture the complexity of activities conducted in laboratories developing gene drive strains. Consideration should be given to how strains are maintained. Population suppression strains can be maintained in a mixed form of transgenic and nontransgenic individuals every generation. Both categories of gene drive application (modification and suppression) present somewhat different hazards in the event of escape. Therefore, each should be considered in the context of whether the unmodified species is present in the vicinity of the facility performing the research and whether pathogens known to be transmitted by the arthropod species are in local circulation, as this will influence the likelihood of local establishment of the engineered gene drive trait or increased disease transmission due to the escape of engineered organisms. Escape of a mixture of engineered gene drive modified and unmodified individuals requires consideration of the phenotypes and progressive changes that might result from each of these types independently but also their possible interaction.

Risk assessment should consider the ecology of the local area where the research facility is located and a determination made as to whether the arthropod species is currently present or not in the local environment, and if not present, whether conditions are suitable for the arthropod to become established.

Given the location, the environment surrounding a research institution includes the local public, and risk assessments should integrate anticipated concerns of community residents and public officials. Development of a Crisis Communication Plan (Seeger 2006) (https://emergency.cdc.gov/cerc/resources/templates-tools.asp) and whether the institution has established processes to engage the public for its immediate implementation should be considered.

Regardless of the gene drive technology employed, ACL-2 containment measures within the current ACG v3.2 remain appropriate if the work to be conducted is at a location where the arthropod species clearly cannot become established based on available evidence (Table 4). If the wild-type arthropod in question is currently not present in the area, gene drive approaches associated with expected mild (population refractory) or robust (population suppression) fitness costs may present a reduced risk of establishment as compared with unmodified arthropods of the same species.

### Research conducted where the arthropod species is not found but could become established

Autonomous gene drives designed to suppress arthropod populations are expected to reduce the likelihood of their establishment in the environment, and arthropods with a suppression phenotype would be considered “disabled” under the ACG v3.2 framework. Such arthropod strains are unlikely to become established in the absence of an indigenous wild-type population unless a high rate of failure of the driving transgene has been observed in experimental evaluations, in which case the nondriving form could become established as a new arthropod population.

Arthropods modified with a transgene(s) that reduces vectorial capacity (probability of the arthropod to successfully transmit a pathogen) may present a similar risk of establishment as do unmodified arthropods unless the drive confers significant reductions in fitness and likelihood of establishment. However, due to the intended effect of such gene drive genetic elements, the resulting phenotype may result in a reduced risk of pathogen transmission relative to the unmodified arthropod population.

For split gene drive and other self-limiting approaches (see Glossary of Terms), one of the transgenes may confer arthropod population suppression or reduce vectorial capacity but have no capacity to drive on its own. These phenotypes may present greater risk of establishment than an autonomous suppression drive (discussed above) due to the potentially rapid population suppression. However, the fate of any nondriving transgene designed to affect vector competence may be difficult to predict with reasonable certainty. The possibility of both driving and nondriving transgenes being lost from a newly established population over subsequent generations should be considered.

### Research conducted where the arthropod species is found

Whether the gene drive is predicted to reduce vectorial capacity or reduce arthropod populations, if the consequences of an escape of these gene drive-containing arthropods is anticipated to be an uncontrolled, unapproved, and potentially irreversible introduction into the environment, this would represent the highest risk level of these three scenarios. Although this would include many autonomous gene drive approaches, even some higher threshold drives may persist if the initial escape is into small, relatively isolated, wild-type populations in which the threshold can be reached. Such considerations (including the possibility of not conducting the research) would/should be informed by the risk assessment.

Split gene drive approaches provide limitations for the ability of the corresponding transgenes to spread into a specific population, and so are predicted to be associated with reduced risk of local establishment. However, these approaches do not protect against the introduction of novel transgenes into a wild-type arthropod population due to unintended intermating with arthropods containing the genetically assorting transgenes. Thus, the phenotype of each transgene must be considered independently as described previously in the ACG v3.2.

Similar concerns may exist for other self-limiting gene drive approaches, in that nondriving transgenes may persist in the environment and may express a harmful novel phenotype. Risks due to failure of any self-limiting mechanisms should also be considered.

### Reducing and managing risks

On completion of a risk assessment, the next step is to evaluate to what extent risks could be mitigated or managed. This requires that the applicant and those conducting the risk assessment consider how potential harms can be avoided, mitigated, and managed per institutional, local, and national requirements. As an example, one might identify measures that could be taken if escape of an arthropod carrying a gene drive led to establishment of an exotic vector in the environment. Consideration should be given to the sensitivity, reliability, and robustness of the monitoring methods to detect such an establishment event and whether such an occurrence could be reversed by current control measures. This requires input by subject matter experts and possibly the expertise of local agencies that are responsible for arthropod control.

### Risk considerations for laboratories exchanging strains

Laboratories conducting research on gene drives may exchange transgenic and wild-type strains among institutions, either in the same country or internationally. It has been recommended that no transfer of reproductively viable arthropod life stages containing gene drive strains be performed between laboratories (Akbari et al. 2015). However, here we highlight that means to ship hazardous microbes have been developed and approved by the International Air Transport Association (2020–2021) and the World Health Organization (2020). Guidance also includes secure containers to contain GM organisms (International Air Transport Association 2020–2021). Shipping and receiving institutions should consider documentation of responsibilities and liabilities with, for example, a memorandum of understanding or material transfer agreement.

The possibility of inadvertent transfer of a gene drive through a strain that is unknowingly contaminated with a gene drive modification in the laboratory should be considered (Benedict et al. 2018). If such a contaminated strain were shipped to a location that does not have the adequate level of containment infrastructure/security in place to contain the gene drive, the strain should be destroyed to prevent escape into the environment.

## Arthropod Containment Levels 2+ Recommendations

ACL-2+ recommendations should be implemented if there is the potential for arthropods to initiate sustained gene drive in the local environment, as determined based on a risk assessment conducted by the lead investigator and reviewed by the local biosafety committee or equivalent. As an example, mosquitoes modified to contain an autonomous gene drive in a location where the same species is present would pose a higher risk for potential spread or persistence due to escape than if the same experiments were conducted in an area where the species is exotic and could not become established (see Risk Assessment section).

If the risk assessment indicates that the potential for establishment or persistence is low to none, the baseline level of containment recommendations for each individual modified strain would remain ACL-2, as indicated in the currently published ACG v3.2 (American Committee of Medical Entomology; American Society of Tropical Medicine and Hygiene 2019). However, even in the absence of gene drive, genetic modifications that increase vectorial capacity due to changes in arthropod viability, survivorship, and/or host range could have the potential to spread or persist in the environment, and therefore ACL-2+ recommendations would be more appropriate. Even unmodified strains may benefit from ACL-2+ recommendations based on specific risk assessment findings. For example, unmodified strains may be invasive, whereby they have the potential to become newly established in a hospitable environment.

### Introduction to ACL 2+ recommendations

ACL-2+ builds on the standard practices, procedures, containment equipment, and facility requirements outlined in the ACG v3.2 and currently recommended for ACL-1 and ACL-2 with inclusion of additional relevant recommendations (Table 5). To determine the appropriate recommended containment level, each row presented in the risk assessment section of Table 5 should be considered individually. Containment should be set based on the highest level selected for any individual row. Importantly, as noted in Table 5, considerations for the potential for sustained gene drive must consider the local environment in combination with the specific transgene architecture, not simply its behavior in controlled laboratory conditions. Essentially, ACL2+ is more stringent than ACL-2 with a particular focus on greater restrictions to laboratory access, better segregation of workspaces, increased strain verification, and more training, but less stringent than ACL-3. Hereunder, we refer to current ACL-2 recommendations where those remain relevant with the phrase “As per ACL-2,” at the beginning of each recommendation for easy reference. We also describe how new and/or adapted recommendations differ between ACL-2 and ACL-2+ as “Notes” below each recommendation, as appropriate. Any examples listed in parentheses should not be considered exhaustive but rather are meant to highlight those that may be most common.

**Table 5.** Overview of Arthropod Containment Levels (ACL 1–4) and Biosafety Containment Levels (BSL 1–4) to Include Arthropod Containment Level-2+ Presented in This Addendum

Risk assessment (see Risk Assessment of Gene Drive Strains section)					
Consideration (not exhaustive)	Appropriate ACL 1–4				
	1	2	2+	3	4
Infection status; Presence or absence, type of pathogen	Up to BSL-1^a^	Up to BSL-2^a^		Up to BSL-3^a^	Up to BSL-4^a^
The species in the local environment	Indigenous species with no change in local fauna or exotic but inviable or transient	Exotic with potential to establish			
Transgenic status	Nontransgenic	Transgenic			
Potential for sustained gene drive^b^	N/A	None to low	Moderate to high		
*Containment (see Arthropod Containment Level 2+ Recommendation section)*					

Category (not exhaustive)	Recommendation for ACL 1–4				
	1	2	2+	3	4
Handling practices	ACL-1 standard handling practices	ACL-2 and BSL-2^a^, limited access, training, signage, containment, and disposal	ACL-2 and BSL-2^a^, restricted access, training, appropriate PPE, signage, SOPs, disposal, containment, record keeping^c^, shipment considerations	ACL-3 and BSL-3^a^, restricted access, training, appropriate PPE, signage, containment, disposal, record keeping^c^	ACL-4 with BSL-4^a^ isolation, training, appropriate PPE, signage, containment, disposal, record keeping^c^
Primary barriers	Species-appropriate containers	Appropriate PPE, containers that prevent escape	Appropriate PPE, containers that prevent escape, methods available for emergency destruction of arthropod^c^	Appropriate PPE, escape-proof containers, pesticide available for emergency use^c^	Appropriate PPE, escape-proof containers, pesticide available for emergency use^c^
Secondary barriers	Not required	BSL-2^a^ facilities, breeding sites, and harborage minimized, pest control	Up to BSL-2^a^ facilities, pest control^c^, other physical containment devices and structural features	BSL-3^a^ facilities, biological safety cabinets, other physical containment devices, pest control^d^	BSL-4^a^ and facility-specific procedures and equipment for arthropod handling while wearing positive pressure containment suite^d^

Adapted from the Arthropod Containment Guidelines v3.2, American Committee of Medical Entomology; American Society of Tropical Medicine and Hygiene 2019. General guidelines for best laboratory containment practices are shown for vector species according to ACL and biosafety level. Indigenous species are those species whose current range includes the research location. All others are considered exotic. Containment guidelines take into account the consequences of escape from a laboratory, in which the arthropod would be (1) inviable as a result of exposure to unfavorable conditions, (2) transient because conditions vary such that the arthropod would die during typical year climate cycle, or (3) has potential for establishment if escaped arthropods could reasonably be expected to persist through a typical climatic year. Arthropod containment specifics for each BSL should always be reviewed in the context of a laboratory-, vector-, and pathogen-specific risk assessment that is based on consultation between the investigator and the appropriate institutional oversight committee(s).

^a^ U.S. Department of Health and Human Services, Public Health Service, Centers for Disease Control and Prevention, National Institutes of Health (2020).

^b^ Evaluations concerning the potential for sustained gene drive must consider the local environment and number of individuals that would have to escape along with the specific transgene architecture.

^c^ Additional restrictions apply for work with arthropods in association with select agents.

^d^ When gene drive strains contain BSL-3 and BSL-4 agents, measures that are recommended for ACL-2+ should be considered for their compatibility with BSL-3 and BSL-4 requirements. At these levels, pathogen containment measures supersede incompatible ACL-2+ recommendations.

ACL, Arthropod Containment Level; BSL, biosafety containment level; PPE, personal protective equipment; SOP, standard operating procedure.

Recommendations outlined must be met in accordance with any pre-existing institutional occupational safety and health regulations.

### Facility design and layout

The manipulation of arthropods modified to contain transgenes with the potential to invade native populations in the local environment necessitates a more stringent facility design than specified by the ACG under ACL-2. Dedicated spaces to be used as insectaries are required to ensure proper containment and rigorously restrict access. Researchers should consult the “NIH Design Requirements Manual” (U.S. National Institutes of Health, Office of Research Facilities (ORF) 2016); specifically appendix O.1. that details considerations for insect facilities. This includes facility design, planning considerations, basic requirements, architectural design, heating, ventilation and air-conditioning design, plumbing, and electrical design.

#### Location of arthropods

Arthropods are located in dedicated rooms, suites, or facilities isolated from unrelated research activities. As specified in the ACG v3.2, these dedicated spaces are referred to as an insectary, even if the arthropods in question are noninsects such as ticks. Increased levels of physical isolation are recommended (separate rooms, suites, and buildings) depending on the potential of the arthropod or transgene to spread in the local environment. If arthropods such as ticks are transported to animal housing for feeding, care must be taken to transport the ticks in a nonbreakable container, preferably in a plastic syringe or a scintillation vial kept inside a humid box (Almazán et al. 2018, Nuss et al. 2017). Vertebrate animals used for tick feeding should be housed in a dedicated room in which the ACL-2+ guidelines recommended here (light-colored walls, no drains, no windows, no equipment other than the vertebrate cages, and fitted with a sticky mat or a double-sided tape inside the door threshold and frame) are employed. To account for all individuals (fed or unfed), ticks should be placed in capsules glued to the animal's back and monitored daily until all are returned to the insectary (Embers et al. 2013, Mateos-Hernández et al. 2020). If ticks are housed in the animal facility for rearing (insectary within animal housing), a dedicated incubator should be used, and the suite should follow the ACL-2+ guidelines already described.

*Note:* This recommendation exceeds ACL-2 guidance that simply recommends a dedicated space for arthropods and exempts ticks from such a dedicated space. Whereas ACL-2 allows for dedicated “closets” or “incubators” to fulfill this requirement, these are not sufficient for ACL-2+.

#### Insectary doors

As per ACL-2, ACL-2+ recommends that the entrance to the insectary is separated from areas that are open to unrestricted personnel through a double-door vestibule that impedes arthropod escape.

#### Insectary windows

Windows directly separating the insectary from the outside environment should ideally not be present; however, if this cannot be avoided, external windows must be sealed and be impervious to breakage. Internal windows that may exist between rooms of a laboratory suite should not allow opening and be resistant to breakage.

*Note:* This recommendation exceeds ACL-2 guidance in that it specifies terms of exterior windows, from “not recommended” to “should ideally not be present.”

#### Interior surfaces

As per ACL-2, ACL-2+ recommends an insectary used to house arthropods (with invasive transgenes or those that are themselves considered an invasive species) is designed, constructed, and maintained to facilitate identification and destruction of arthropod escapees. The interior walls, ceiling, and flooring should be light colored, nonwooden, smooth, and uncovered, so that escaped arthropods can be visually seen and tracked for recapture and/or destruction. Gloss finishes, ideally resistant to chemical disinfectants and fumigants, are recommended. Where flying arthropods are present, ceilings are at a height to allow visual detection of escapees.

#### Vacuum systems

As per ACL-2, ACL-2+ recommends if a central vacuum system is installed, suitable barriers/filters appropriate for the arthropod being handled are in place to prevent arthropod escape. Institutional regulations on facility safety requirements must be considered.

#### Source and harborage reduction

As per ACL-2, ACL-2+ recommends harborage and breeding areas are eliminated.

#### Equipment and supply storage

As per ACL-2, ACL-2+ recommends equipment and supplies not required for operation of the insectary and/or research activities should not be located or stored in the insectary. Equipment and supplies that are removed from the insectary must be thoroughly inspected before removal to ensure that arthropods are not present.

#### Plumbing and floor drains

As per ACL-2, all existing drains are fitted with screens/filters sized to prevent all life stages of the arthropod from escaping into the sewer.

#### Electrical fixtures

As per ACL-2, ACL-2+ recommends all penetrations of walls, floors, and ceilings are minimal and are sealed/caulked. Ideally, all light fixtures are flush with the ceiling, sealed, and accessed from above.

#### Sink

As per ACL-2, ACL-2+ recommends arthropod escape through plumbing should be prevented by ensuring sink drains are plugged/covered when not in use (see also Plumbing and floor drains section).

#### Illumination

As per ACL-2, ACL-2+ recommends illumination should be appropriate for stock maintenance and should not compromise containment, impede vision, or hinder the enforcement of safety procedures in the laboratory. Lighted or darkened openings that attract escaped arthropods should not be present.

#### Heating, ventilation, and air conditioning

All ventilation ducts are fitted with screens/filters sized to prevent the escape of arthropods. Although directional airflow in the insectary (inward) may be appropriate in some circumstances, in other cases this might elicit arthropod behavioral cues that elicit movement toward the insectary exit.

*Note:* This recommendation differs from ACL-2 in that ACL-2+ recognizes that directional airflow, although important for pathogen containment, may not be an effective arthropod containment measure.

#### Facility compliance monitoring

All relevant responsible parties (principal investigator [PI], facility managers, oversight committees, and regulatory authorities) should verify that the design and operational parameters of the facility have been met before the rearing of nonendemic arthropods, manipulating invasive arthropods, or those modified to contain invasive transgenes. This can be achieved through the rearing and maintenance of unmodified arthropods during a probationary period to identify any defects or problems in facility design/integrity. Although the facility should be formally evaluated annually for compliance to ACL-2+ by biosafety professionals, more frequent inspections by the PI or responsible designee should be conducted based on the conclusions of the risk assessment. For example, more regular inspections of the facility and work operations are performed (monthly, weekly, and/or daily) to ensure both the physical integrity of the insectary (seals, caulking, and screens) and the proper work habits of laboratory personnel.

*Note:* This recommendation exceeds ACL-2 guidance, in that ACL-2+ calls for more frequent inspections of the facility and work practices.

### Procedures: access/security

#### Security

Insectary entrances should be locked 24 h a day. Access should be regulated by a key fob, card reader, or similar methods to enable individual tracking of authorized personnel. The use of hard keys is not recommended, as these are easily duplicated. Consideration should be given to security cameras as needed based on risk assessment, both inside insectaries, as well as outside the facility/building/structure to remotely monitor facility integrity, operational work practices, and entry by unauthorized persons. If emergency exits are present within the insectary, procedures for how these may be utilized without compromising containment should be specified. The installation of audible alarms that sound when any such emergency exits are opened is recommended.

*Note:* This recommendation exceeds ACL-2 guidance, in ACL-2 guidance as ACL-2 does not present specific security recommendations.

#### Access restrictions

Insectary and/or laboratory access is limited to the fewest number of persons possible. Visitors and guests are strongly discouraged from entering insectaries and/or laboratories while active work is occurring. Custodial personnel do not enter the insectary and/or laboratory. Routine cleaning is performed only by trained laboratory staff.

*Note:* This recommendation exceeds ACL-2 guidance, as ACL-2 allows access to all trained personnel and accompanied guests and does not exclude custodial personnel.

#### Notification and signage

Visitors to the laboratory are always accompanied and should be made aware of (1) the presence of arthropods modified with transgenes capable of gene drive, (2) any potential hazards associated with their escape, and (3) what actions might be needed in the event an escaped arthropod(s) is/are identified. Appropriate signage should list all species handled within the insectary and be updated whenever new species are introduced. Such hazard warning signs typically identify the arthropod species, the presence and type of invasive transgene, the name and telephone number of the responsible person(s), and indicate any special requirements for entering the insectary and/or laboratory.

*Note:* This recommendation differs from ACL-2 in that it adds language associated with invasive transgenes.

#### Prevention of accidental dispersal outside the research laboratory by attachment on persons

Physical barriers (overlapping sheets, screens, air curtains, etc.) at entrances/exits of the room or suite where arthropod handling occurs should be considered. Any personal protective equipment (PPE) (laboratory coats/gowns/hairnets) should not leave the ACL-2+ laboratory or the facility where they are donned/doffed. Laboratory personnel should visually inspect all attire for attached arthropods before leaving the insectary and/or laboratory. Staff are instructed to inspect outer clothing for visual detection of arthropods and are trained to visually identify arthropods in all life stages. Cloth gowns should be laundered routinely to mitigate continual arthropod attachment over subsequent days of use. For this reason, especially where laundering services at the research facility may be absent, disposable gowns are preferred and should be discarded as laboratory waste when signs of wear appear. If gowns are to be laundered, disinfection must occur first. For crawling arthropods, the use of measures such as sticky pads or tape at room thresholds is recommended.

*Note:* This recommendation differs from ACL-2 in that ACL-2+ adds language associated with prevention of accidental dispersal of crawling arthropods.

### Procedures: handling/manipulation

Recommendations outlined for laboratory personnel safety must be met in accordance with any pre-existing institutional occupational safety and health regulations.

#### Procedure design

Procedures are in place to prevent arthropod escape during all manipulations with arthropods containing invasive transgenes. Procedures should be prevalidated by rearing and handling unmodified arthropods before implementation and evaluating the effectiveness of the containment measures. Procedures should be developed, documented, and archived in hard copy for use in training study staff.

*Note:* This recommendation exceeds ACL-2 guidance, as ACL-2+ calls for procedure prevalidation.

#### Routine decontamination

All equipment and work surfaces in the insectary are routinely inspected for the presence of arthropods (all life stages) and disinfested with an effective treatment such as 70% ethanol or soapy water. Cleaning equipment (brooms, mops, etc.) should be labeled and dedicated to each specific containment facility (insectary and laboratory).

*Note:* This recommendation exceeds ACL-2 guidance, as ACL-2+ calls directly for routine inspection of equipment and work surfaces for the presence of arthropod material in addition to regular disinfestation.

#### Special arthropod handling containers and areas

As per work with infected arthropods, escape into the laboratory area of arthropods containing invasive transgenes must be prevented. A dedicated area for handling arthropods with invasive transgenes is present in the insectary. This is preferably a separate enclosed cubicle, screen room, or other enclosed space. Additional physical barriers (*e.g.*, glove box) or procedures (low temperature room) may be required depending on the risk assessment.

*Note:* This recommendation exceeds ACL-2 guidance, as ACL-2+ calls for elevated handling protocols regardless of infection status.

#### Isolation of arthropods

Procedures should be in place to prevent the contamination of wild-type/unmodified arthropod strains with arthropods containing transgenes capable of gene drive. Arthropods containing potentially invasive transgenes should be held and manipulated in a room, suite, walk-in chamber, or facility that is separate from the location housing of reproductively compatible arthropods containing noninvasive transgenes or unmodified arthropods. The amount of separation (number of intervening rooms and/or physical distance) should be proportional to the invasiveness of the transgene and risk of spread and likelihood of distributing stocks to other laboratories. Contamination between arthropods containing different transgenes capable of gene drive should also be prevented by appropriate containment of all the arthropod stages.

*Note:* This recommendation exceeds ACL-2 guidance, as ACL-2+ calls for the separation of strains containing transgenes capable of gene drive from other arthropods.

#### Safe transport in the laboratory

Containers, cages, jars, pans, and other equipment used to house arthropods will be shatter proof and screened or covered with mesh or a lid sufficient to prevent escape. All arthropods with invasive or potentially invasive transgenes are collected, labeled, transported, and processed in a manner that contains and prevents their escape. Transfer of arthropods between manipulation and holding areas will be in nonbreakable secure containers. Transport of unmodified arthropods and/or those containing noninvasive transgenes into areas used for the rearing and/or manipulation of invasive transgenes is considered to be one way and should not return to other spaces in the insectary.

*Note:* This recommendation exceeds ACL-2 guidance, as ACL-2+ calls for elevated handling protocols for circumstances (gene drive transgenes) other than with infected arthropods.

#### Primary container identification and labeling

As per ACL-1, ACL-2+ recommends all containers are clearly marked to easily distinguish individual transgenic arthropod strains.

#### Primary container cleaning and disinfestation

Procedures are in place regarding the devitalization of pans, cages, or other containers used to hold arthropods modified with invasive transgenes. To reduce the risk of mixing arthropods with and without invasive transgenes by having different methods for disposal, a single waste stream involving autoclaving, incineration, or other appropriate decontamination methods of all primary containers should be used. Use of disposable containers and materials for rearing arthropods with invasive transgenes is recommended when practical.

*Note:* This recommendation exceeds ACL-2 guidance, as ACL-2+ calls for a laboratory to use a single waste stream procedure and consideration of disposable containers.

#### Disposal of arthropods

All life stages of arthropods must be killed before entering the waste stream by freezing, boiling, mechanical disruption, bleach/ethanol treatment, or other methods based on risk assessment. Procedures should specify validated contact times/concentrations/temperatures. All arthropod remains should be collected in a solid waste stream during transport and delivery to an autoclave or incineration.

*Note:* This recommendation exceeds ACL-2 guidance, as ACL-2 calls for such elevated handling protocols only with infected arthropods and does not comment on disposal requirements for killed material.

#### Disposal of consumable supplies

Consumable supplies such as Petri dishes, vials, and primary containers that were used to handle, rear, and/or house arthropods as well as cloth and disposable gowns may be considered regulated waste according to some institutional policies and thus should be disposed of according to risk management processes outlined by those institutions. This may include that material is decontaminated (autoclaved) before disposal.

*Note:* This recommendation exceeds ACL-2 guidance, as ACL-2 provides no specific direction regarding disposal of consumable supplies.

#### Prevention of accidental dispersal outside the research laboratory through sewer system

Care should be taken to not disperse any arthropod material into the drainage. No material that may have been associated with arthropod rearing (water, paper, and soil) is disposed through the sewer unless it is devitalized. Devitalization techniques could include heat or chemical treatment, freezing, or filtration, as appropriate, and should be based on risk assessment. Consideration should be given to the collection and sterilization of all wastewater before disposal through the general sewer system.

*Note:* This recommendation differs from ACL-2 in that ACL-2+ replaces language associated with pathogen-infected arthropods with concerns relevant for invasive transgenes, the direct specification of possible devitalization techniques, and in calling for the collection/sterilization of wastewater used in conjunction with invasive transgenes when risk of harm to protection goals is high.

#### Movement of equipment

As per ACL-2, ACL-2+ recommends all equipment used in conjunction with arthropods containing invasive transgenes must be appropriately disinfested before transfer between rooms within the insectary, and before removal from the insectary.

#### Sterilization equipment

An autoclave is available and, whenever possible, is located within the insectary itself. If the autoclave is present within the building, but outside of the contained insectary, procedures are in place to prevent the escape of arthropods during transport, either viable stages improperly placed into the waste stream, or escaped arthropods that found refuge on or about waste containers and/or laboratory personnel attire. If an autoclave is not available, other alternative disinfestation practices may be recommended by the risk assessment.

*Note:* This recommendation exceeds ACL-2 guidance, as ACL-2+ provides additional direction regarding developing procedures for transporting waste before sterilization.

#### Laboratory sharps

As per ACL-2, ACL-2+ recommends that the use of disposable sharps should be minimized, with any sharps either decontaminated after each use (ethanol or flame sterilization) or disposed of in puncture-proof containers or as mandated by institutional policy for hazardous waste. This is a particular concern for small arthropods such as ticks that can crawl up and hide in research instruments such as forceps.

#### Pest exclusion program

As per ACL-1.

### Procedures: monitoring/training

#### Institutional biosafety committee and institutional animal care and use committee approvals

Institutional biosafety committee (IBC) (or similar biosafety oversight body) review and approval of the research are required before initiating activities with transgenic arthropods. All institutional oversight bodies should be provided with adequate information concerning the nature of any transgene or combination of transgenes that might be invasive or that has the potential to persist in the environment, per the institutional protocol submission procedures.

*Note:* This recommendation differs from ACL-2 in that ACL2+ adds language associated with invasive transgenes.

#### Safety manual

A site-specific safety manual is prepared, approved by the IBC and/or other institutional review entities, and adopted. This manual should specify how containment is maintained, including standard operating procedures (SOPs) for waste disposal, escaped arthropod monitoring, facility maintenance, entry/exit protocols, access requirements, any other information necessary to inform personnel of the methods for safe maintenance and operation of the insectary, and notification plan in the event of a potential escape. Some of the elements to be included in the safety manual are described in the Training, Monitoring for Unintentional Arthropod Escape, Escaped Arthropod Handling, Escaped Arthropod Reporting, Record Keeping of Escaped Arthropods, and Personal Protective Equipment sections. If the institution does not have formal review committees, the person responsible for the research should request a review of all procedures, safety precautions, and facilities by colleagues who are experts in the field.

*Note:* This recommendation exceeds ACL-2 guidance, as ACL-2+ cites additional SOPs directly and calls for an external review of these SOPs if a local committee is not present.

#### Training

Laboratory personnel are advised and trained on special hazards, practices, and procedures outlined for handling arthropods as described in the safety manual. Adherence to established safety and handling procedures and policies is made a condition of employment and is part of the annual performance review, if applicable, of every employee with responsibility in the insectary. Personnel receive annual updates and additional and/or refresher training as necessary for procedural or policy changes. Personnel records of all completed training sessions by date are maintained. Proficiency in all required laboratory procedures and work practices using unmodified arthropods should be assessed and documented before any individual beginning work with arthropods containing engineered transgenes capable of gene drive, with level of training and experience required proportional to the invasiveness of the transgene.

*Note:* This recommendation exceeds current ACL-2 guidance, as ACL-2+ requires study staff to demonstrate proficiency in all laboratory practices before work with invasive transgenes.

#### Monitoring for unintentional arthropod escape

An effective arthropod monitoring program is established. An effective program may include arthropod trapping (oviposition traps, ground-level flea traps, double-sided tape or oil-filled channels surrounding tick colonies, or light traps for mosquitoes) and surveillance (regular inspections of walls, ceilings, and screens). Counting of individual arthropods may be integrated in the laboratory monitoring program when/if such a practice is standard for the arthropod (*e.g.*, ticks). Procedures should specify the frequency of surveillance activities (*e.g.*, all personnel inspect upon entry/exit) and when traps are checked. As the risk associated with an invasive transgene increases, these frequencies should be greater as appropriate. Procedures should specify how escaped arthropods can be identified as containing an invasive transgene or not (such as the presence of unique, visible, or molecular markers) and when such identification is performed.

*Note:* These recommendations exceed those specified under ACL-2. Specific enhancements for ACL-2+ are additional directions concerning monitoring activities other than trapping, calls for the ability to identify the escaped arthropod's genotype, and increased monitoring activities in proportion to the invasiveness of the transgenes under study.

#### Escaped arthropod handling

Any arthropods found outside of primary containment (*e.g.*, cages, dishes, jars, or pans) should be killed upon discovery. Species-appropriate tools for kill/capture (vials of ethanol, nets, racket zappers, fly swatters, etc.) should be easily accessible. Procedures should be in place for notifying laboratory personnel and temporary suspension of investigations while a recapture search is in progress, and to identify the source of escapees. Procedures are in place that dictate under what conditions work may resume if an escaped arthropod is not located (see Escaped Arthropod Reporting section).

*Note:* This recommendation differs from ACL-2 guidance, as ACL-2 permits the recapture of escaped arthropods but ACL-2+ does not.

#### Escaped arthropod reporting

Procedures are in place for reporting escaped arthropods with invasive transgenes, including points of contact for the person(s) responsible for the research and/or any mitigating actions. Procedures that result in the escape of arthropods from primary containment vessels must be reported immediately to the individual responsible for ensuring that appropriate and documented action is taken to mitigate the escape. Follow-up evaluation of all relevant protocols and procedures should be undertaken to prevent similar events, along with retraining of insectary personnel, as necessary.

*Note:* This recommendation differs from ACL-2 in that it adds language associated with invasive transgenes.

#### Record keeping of escaped arthropods

As per ACL-3, records of escaped arthropods are maintained and reviewed on a routine basis, review schedule to be relevant to quantity and/or frequency of handling the arthropod. Any evidence of escape (to include within the laboratory if the insectary is located in a suite) should be included during review of practices and procedures and refresher training on laboratory-specific SOPs for research personnel prescribed as needed. Procedures should also allow the differentiation of laboratory-reared arthropods from wild-type arthropods of the same species that may be identified through the monitoring program. This is particularly relevant when risk assessment includes the requirement for monitoring outside the insectary (in clean corridors or outside the facility). As many trapping mechanisms are destructive due to fans or mass collection chambers, less destructive methods such as sticky cards may be preferred for the unambiguous identification of individual arthropods.

*Note:* This recommendation exceeds ACL-2 guidance, as ACL-2 specifies only those records of “exterior captures are maintained” and no specific guidance is provided as to how or when these records are reviewed.

#### Personal protective equipment

Recommended PPE should minimize the amount of exposed skin and should be evaluated as part of the site-specific risk assessment. Although arthropods with invasive transgenes are unlikely to pose a direct hazard to laboratory personnel, the use of gowns/laboratory coats/hair nets can facilitate the detection of escaped arthropods and prevent the accidental removal of viable life stages on clothing. Similarly, the use of gloves may be appropriate for handling small arthropod life stages such as eggs. Note: PPE should not leave the contained space.

*Note:* This recommendation differs from ACL-2 in that it adds language associated with invasive transgenes.

### Other procedural considerations

#### Containment director

Depending on the scale and scope of the research to be conducted with the arthropod and the site-specific risk assessment, a containment director (CD) that oversees daily operations and the physical integrity of the facility should be assigned. The CD should maintain and implement the SOPs of the facility and is responsible for regular review and updating of these procedures. Specific roles of the CD may also include monitoring facility access, responding to emergency events, monitoring, and addressing physical repairs, training staff, and maintaining insectary records to include institutional biosafety and other regulatory permits, strains maintained, incoming and outgoing shipments, authorize staff, training proficiency, facility maintenance, and staff contact information (United States Department of Agriculture; Animal and Plant Health Inspection Service Plant Protection and Quarantine 2002).

*Note:* This recommendation exceeds ACL-2 guidance, as ACL-2 makes no specific recommendations concerning a CD.

#### Large-cage indoor trials

Laboratory trials may lead to larger scale experiments, with potential requirements of manipulating greater number of arthropods in large enclosed cages and/or walk-in structures. In such cases, monitoring of the physical integrity of the experimental environment (screen mesh surrounding cages, sealants, and locks for walk-in structures) should be performed at increased frequencies related to trial frequencies and risk assessments. Procedures may require increased training associated with workforce expansion, the designation of additional oversight staff, and increased monitoring of scale-up activities (Adelman et al. 2017).

*Note:* This recommendation exceeds ACL-2 guidance, as ACL-2 makes no specific recommendations concerning large-scale trials.

#### Emergency planning

Procedures should be in place to stop research activities rapidly and securely with arthropods containing invasive transgenes in an event of an emergency within the insectary, laboratory, and/or facility. Localized events such as a burst pipe or small fire might require a simple suspension of work or other restrictions on manipulations until containment and safe operations are restored. More catastrophic events might require procedures to rapidly devitalize all arthropods containing invasive transgenes. This could include fumigation, insecticide treatment, or increasing the temperature of environmental chambers housing arthropods above the lethal temperature, among other possible measures. The availability and reliability of any redundant systems, including backup generators or portable autoclaves, should be periodically evaluated.

*Note:* This recommendation exceeds ACL-2 guidance, as ACL-2 makes no specific recommendations concerning emergency planning.

#### Shipping

When shipping a gene drive strain to countries where the arthropod species is present, and particularly to countries where pathogens of human or animal disease are known to be vectored by these arthropod species, rigorous packaging, shipment, and customs clearance must be assured. Laboratory receipt logs, including chain of custody records, are recommended to be integrated within processes of the research institutions(s), shipping courier, and/or state and national regulatory policies.

Appropriate permits are in place for intended international exportation/importation as well as intracountry transfers per institutional and national regulatory policies. Shipping containers conform to best practices and requirements of the shipper and the regulatory body issuing shipment permits (IATA 2020–21). Typically, viable arthropods should be placed within multiple nested containers, with at least one hard nonbreakable liquid impermeable container. The number of arthropods in each container should be noted and monitored upon receipt. Shipping containers are packed/unpacked only within the secure environment of the insectary. When shipping arthropods with invasive transgenes, prescreening and postshipping procedures should be in place to ensure the integrity and authenticity of the material. Even for laboratories receiving nontransgenic arthropods, the recipient laboratories should be informed that invasive transgenes are present in the same facility from which the material was shipped and should be given protocols or other instructions (phenotype, PCR) on how to detect the presence of a driving transgene upon receipt.

Receiving procedures should be in place and include recording date of receipt at the receiving laboratory and contingencies concerning breaches in interior containers that might arise during transit and are only discoverable upon opening. When shipping unmodified arthropods or those without invasive transgenes, precautionary prescreening for the presence of any invasive transgenes in use in the shipping laboratory is required.

*Note:* This recommendation exceeds ACL-2 guidance, as ACL-2 makes no specific recommendations concerning strain validation before shipping.

## Appendix

### Appendix A1. Addendum 1 Drafting Committee Members

Achee, Nicole L., University of Notre Dame, Notre Dame, IN

Adelman, Zach N., Texas A&M University, College Station, TX

Benedict, Mark Q., Centers for Disease Control and Prevention, Atlanta, GA

Dotson, Ellen M., Centers for Disease Control and Prevention, Atlanta, GA

Duman-Scheel, Molly, Indiana University School of Medicine, South Bend, IN

Gulia-Nuss, Monika, University of Nevada, Reno, NV

Tarimo, Brain, B., Ifakara Health Institute, Bagamoyo Office, Tanzania

### Appendix A2. Addendum 1 Drafting Process

The drafting committee was appointed by ACME in August of 2020. See Intent section of the addendum for further details. The completed draft was circulated to all ACME members in February 2021.
